# The human proteome – a scientific opportunity for transforming diagnostics, therapeutics, and healthcare

**DOI:** 10.1186/1559-0275-9-6

**Published:** 2012-07-03

**Authors:** Marc Vidal, Daniel W Chan, Mark Gerstein, Matthias Mann, Gilbert S Omenn, Danilo Tagle, Salvatore Sechi

**Affiliations:** 1 Dana-Farber Cancer Institute, Boston, MA, 02215, USA; 2 The Johns Hopkins University School of Medicine, Baltimore, MD, USA; 3 Yale University, Connecticut, 06511, USA; 4 Max Planck Institute for Biochemistry, Martinsried, D-82152, Germany; 5 University of Michigan, Ann Arbor, MI, USA; 6 National Institute of Neurological Disorders and Stroke, Bethesda, MD, 20892, USA; 7 National Institute of Diabetes and Digestive and Kidney Diseases, National Institutes of Health, 6707 Democracy Blvd. Rm 611, Bethesda, MD, 20892-5460, USA

## Abstract

A National Institutes of Health (NIH) workshop was convened in Bethesda, MD on September 26–27, 2011, with representative scientific leaders in the field of proteomics and its applications to clinical settings. The main purpose of this workshop was to articulate ways in which the biomedical research community can capitalize on recent technology advances and synergize with ongoing efforts to advance the field of human proteomics. This executive summary and the following full report describe the main discussions and outcomes of the workshop.

## Executive summary

A National Institutes of Health (NIH) workshop was convened in Bethesda, MD on September 26–27, 2011, with representative scientific leaders in the field of proteomics and its applications to clinical settings. The main purpose of this workshop was to articulate ways in which the biomedical research community can capitalize on recent technology advances and synergize with ongoing efforts to advance the field of human proteomics.

Proteins are the major components of biological networks and molecular machines, and proteins are the targets for the large majority of drugs available today. Participants in this Workshop recognized that a deeper knowledge of the human proteome could help fill the gap between genomes and phenotypes, transform the way we develop diagnostics and therapeutics, and thereby enhance overall biomedical research and future healthcare. The Human Genome Project and its many follow-on initiatives, including the HapMap and ENCODE, together with advances in protein sciences, have provided a foundation for proteomic technologies and informatics resources. Several major initiatives are already moving toward deep characterization of the human proteome, including the antibody-based Human Protein Atlas, the NIH Common Fund Protein Capture Reagents, the mass spectrometry-based Peptide Atlas and Selected Reaction Monitoring (SRM) Atlas, and the Human Proteome Project organized by the Human Proteome Organization. Several leading laboratories have demonstrated that about 10,000 protein products, of the about 20,000 protein-coding human genes, can be identified and quantified in a single experimental specimen; this figure may represent nearly the complete complement of proteins actually expressed in a single cell type. In yeast the complete expressed proteome has been identified. Even though a more comprehensive characterization of the dynamic aspect of the proteome will require further technology development, it is a disruptive concept that almost all of the primary products of the genome can be detected at the protein level in one single experiment.

The Workshop was organized in five sessions: (1) protein networks; (2) integrating proteomics with other omics; (3) quantitative proteomics by exploratory and targeted methodologies; (4) study design and statistical challenges in clinical proteomics; and (5) proteomic technologies in a clinical setting. Sessions 1–3 constituted a main theme on systems biology; sessions 4–5 represent a theme on strategies for clinical proteomics. The full agenda is at http://www3.niddk.nih.gov/fund/other/HumanProteome2011. This executive summary and the following full report describe the main discussions and outcomes of the workshop.

### Protein networks: toward a comprehensive wiring diagram of human cells

The interactome network of cells is the complete set of macromolecular interactions that take place between genes and gene products; it is mostly mediated by proteins. Pull-down of diverse protein complexes and sequencing of the components, plus other direct measurements of protein-protein, protein-nucleic acid, and protein–lipids interactions now make it feasible to create wiring diagrams for systems biology. The capability of quantifying the main gene products and providing information on post-translational modifications and splice variants of proteins addresses the dynamic nature of the networks. Experimental and natural perturbations of cultured cells and of whole organisms can then reveal connectivity and can test hypotheses of the blueprint and dynamic regulation of phenotypes. Informatics tools such as Cytoscape and databases such as BioGRID provide means of visualizing pathways, networks, and interactomes.

### Integrating proteomics with other omics

Detailed integration of data and knowledge from multiple omics technology platforms is essential for building and understanding the pathways from genome to phenotypes and the influence of environmental and behavioral variables. The influence of allelic variants, splice variants, and post-translational modifications must be assessed in combined analyses of mRNA and protein abundance and response to perturbations. Single nucleotide polymorphisms and alternative splicing can influence sites of post-translational modifications, magnifying their downstream effects. Linking gene expression, protein expression, and metabolomics has become an attractive approach, facilitated by new bioinformatics tools.

### Quantitative proteomics by exploratory and targeted mass spectrometry methods

There has been stunning progress in mass spectrometry-based proteomics, with new technologies and combinations of instrumentation for bottom-up peptide analysis, top-down protein analysis, and targeted quantitative analysis of proteotypic peptides of selected proteins. The equipment and reagent sector for proteomics is a major economic engine. These advances enable much more potent approaches for biomarker development and protein targeting with therapeutic agents. In contrast to recent studies limited to the most abundant dozens or hundreds of proteins in a biological specimen, current experiments are identifying, with a false discovery rate of one percent, and quantifying approximately 10,000 proteins from different genes in human cell lines, for example. Such deep analyses permit direct comparison with deep sequencing of the transcriptome, as well as with protein expression based on immunohistochemistry, as documented in the Human Protein Atlas.

### Study design and statistical challenges in clinical proteomics

For the past decade, many of the individual institutes of the NIH have supported programs and projects to generate potential protein biomarkers for early or more specific diagnosis or prognosis of a wide array of diseases. There are many complex challenges in developing such omics-based tests. Heterogeneity of etiology, pathogenesis, and responses to therapy among patients with identical diagnoses, is common. Knowledge of mechanisms, mediated by pathways and networks, is fundamental to moving beyond statistical correlation as a basis for biomarker development. Integration of data across multiple levels of omics analyses should facilitate such knowledge development. Several general recommendations for biomarker discovery projects were also made during the discussion. Participants emphasized the importance of specifying as early as possible in the process the intended clinical use, and the importance of proper study design in order to avoid introducing bias. Finally, it is important for translational scientists to understand the long path of discovery, confirmation, validation, clinical trials, and FDA approval to establish test validity and utility and gain reimbursement of the laboratory service.

### Proteomic technologies in a clinical setting

The late-stage translation of proteomic technologies and protein biomarker candidates into clinical tests requires specification of the intended clinical use, sufficient evidence in preliminary studies to support the investment for a large-scale validation trial, demonstration of reliable test performance characteristics, and sufficient clinical benefit to gain acceptance by the clinical community. It is counterproductive to try to short-circuit these complex steps, especially in the absence of a strong biological foundation for the biomarker candidate or panel of biomarkers. The development of a roadmap for the translation of proteomics technologies into clinical settings will require close collaboration between researchers, industries, regulators, clinical chemists and clinicians, including private-public partnerships to leverage existing NIH programs. Such a partnership would accelerate the development of clinically useful technologies and biomarkers and make a significant impact to fulfill the unmet clinical needs for patients’ personalized health care.

## Conclusions

### Human proteome networks in health and disease - A major scientific opportunity

A session within the Workshop was dedicated to discussion of potential scientific opportunities and identification of the most compelling ideas for future developments in the field of proteomics. Several potential concepts were considered in the context of the presentations that were made by the speakers. The participants reached consensus that a special opportunity exists at this time for utilizing modern proteomics to link data from multiple levels of omics technologies and build wiring diagrams of human cells and tissues through interactome networks and related phenomena. The focus on protein interactome networks would be salient for all disease processes and would yield a stronger foundation for the many NIH institute-specific programs seeking a more effective translation to biomarker development.

## Full report of the NIH workshop on the human proteome

A National Institutes of Health (NIH) workshop was convened in Bethesda MD on September, 26–27, 2011 with representative scientific leaders in the field of proteomics and its applications to clinical settings. The main purpose of this workshop was to articulate ways in which the biomedical research community can capitalize on recent technology advances and synergize with ongoing efforts to advance the field of human proteomics.

Proteins are the major components of biological networks and molecular machines, and proteins are the targets for the large majority of drugs available today. Participants in this Workshop recognized that a deeper knowledge of the human proteome could help fill the gap between genomes and phenotypes, transform the way we develop diagnostics and therapeutics, and thereby enhance overall biomedical research and future healthcare. The Human Genome Project and its many follow-on initiatives, including the HapMap and ENCODE, together with advances in protein sciences, have provided a foundation for proteomic technologies and informatics resources. Several major initiatives are already moving toward deep characterization of the human proteome, including the antibody-based Human Protein Atlas, the mass spectrometry-based Peptide Atlas and Selected Reaction Monitoring (SRM) Atlas, and the Human Proteome Project organized by the Human Proteome Organization [1-4]. Leading laboratories have demonstrated that protein products of up to ~10,000 of the ~20,000 protein-coding human genes can be identified and quantified in a single experimental specimen [5-7]; this figure may represent nearly the complete complement of proteins actually expressed in a single cell type. In yeast the complete set of expressed proteins has been identified. It is a disruptive concept that the proteome can now be analyzed comprehensively and that all of the primary protein products of the genome can be detected.

The Workshop was organized in five sessions: (1) protein networks; (2) integrating proteomics with other omics; (3) quantitative proteomics by exploratory and targeted methodologies; (4) study design and statistical challenges in clinical proteomics; and (5) proteomic technologies in a clinical setting. Sessions 1–3 constituted a main theme on systems biology; sessions 4–5 represent a theme on strategies for clinical proteomics. The full agenda is at http://www3.niddk.nih.gov/fund/other/HumanProteome2011. The following report describes the main discussions and outcomes of the workshop.

### Protein networks: Toward a comprehensive wiring diagram of human cells (Marc Vidal, Dana-Farber Cancer Institute, chair)

Understanding the detailed mechanistic paths between genotypes and phenotypes is one of the most important goals of biology, and critical in the quest for new therapeutics. Complex genotype-to-phenotype relationships exist in common disorders and traits, but even Mendelian disorders are complicated by phenomena such as incomplete penetrance, variable expressivity, pleiotropy and modifier genes. Recent advances in genome biology have revealed extremely complex links between genotypic modifications and phenotypic changes in cancers, for example. No single phenotype will be fully explained by simple changes in any single gene, because gene/environment interactions and perturbations of biological systems and cellular networks, not single proteins, underlie genotype-phenotype relationships [8]. Vidal defined the “interactome network” of cells as the complete set of macromolecular interactions that can take place between genes and gene products, including protein-protein, protein-DNA, protein-RNA, DNA-DNA, RNA-RNA, enzyme-substrate and post-translational modification interactions. Maps of such macromolecular interactions generated at the scale of the whole proteome will be necessary, although still not sufficient, to fully understand biological complexity. He characterized the current state-of-the-art on interactome networks as similar to the exciting time of the late 1990s for the Human Genome Project. He concluded that the community is ready to produce a “systematic, unbiased, freely available wiring diagram for systems biology” on which to add logical and dynamic relationships. Proteomics is needed to inform those relationships.

Suzanne Gaudet, Dana-Farber Cancer Institute, presented “Predicting phenotypes: quantities and dynamics in proteomics”. Because of complex systems properties that underlie most biological processes, identical genotypes can give rise to very different phenotypic outcomes. In her example, HeLa cells, when treated with the apoptosis-inducing ligand TRAIL, produce two strikingly different populations of cells: 80% die and the remaining 20% survive [9]. Cells that survive this treatment are capable of generating the same bi-modal response when treated a second time. She demonstrated how this variability is mediated biochemically; computer-generated, model-based simulations were able to recapitulate this behavior. In the case of apoptosis, appropriate measurements for a dozen proteins accurately predict the phenotypic outcome of such cell perturbations [10]. Biological experiments over many years and from many laboratories have led to a predictive wiring diagram of this specific biological process. For more general proteome-scale wiring diagram maps, three major components are needed: i) network information on binding partners and biochemical reactions; ii) quantitative information on protein levels, protein affinities and reaction rates; and iii) biosensors to measure response dynamics *in situ*.

Kara Dolinski, Princeton University, presented “Systematic knowledge capture and representation: the Biological General Repository for Interaction Datasets (BioGRID)” [11]. She and her colleagues are tackling the challenge of generating cellular wiring diagrams by collecting and curating published information on macromolecular interactions from both small-scale bottom-up approaches and large-scale proteome-wide mapping enterprises. She presented numbers of downloads and volume of traffic on the BioGrid website that summarizes information so far for yeast and for human biology. Dolinski highlighted exciting prospects for visualization of interactome networks derived from curating literature to generate Bayesian models of disease-specific networks. BioGrid and other databases have become critical for further development of interactome network model-based systems biology. Databases such as the ProteomeXchange, that have emerged as a point of connection for the mass spectrometric proteomic data repositories, should play a key role in the development of a comprehensive wiring diagram of human cells. However, a broad strategy for sustained support of key data resources is needed.

### Integrating proteomics with other omics (Mark Gerstein, Yale University, chair)

Given the scale of other datasets, particularly those derived from next-generation sequencing, much added value is achieved from integrating proteomics datasets with other data. Four key themes for such data integration were identified.

a) *“Direct Integration of mRNA Gene Expression and Protein Abundance Datasets”.* Gerstein described two forms: first, a simplified context for the past decade comparing levels of mRNAs and their corresponding proteins and changes in those levels after a perturbation in a time-course experiment or with successive measurements of clinical specimens; and, second, a more elaborate future context in relation to allelic expression, comparing maternal and paternal alleles both for gene expression and protein abundance using the exact sequences that come from mass spectrometry or transcriptome sequencing [12-14]. The "future case” allows for the examination in detail of the effects of specific mutations on gene expression, using the maternal and paternal alleles as perfectly matched controls. For quantitative proteomics, both for the simpler case of comparing molecular concentrations and the allelic case, one would need the protein abundance sets, preferably including post-translational modifications and splice variants, precisely matched against RNA-Seq sets.

b) *“Connecting Proteomics Data to the Huge Amount of Variation Data”.* Joel Bader, Johns Hopkins University, addressed the idea of connecting proteome data, particularly in the form of networks, with the huge amount of variation data coming from personal genomic sequencing. Other participants also emphasized the importance of connecting the complex aspects of proteins to the variation data. In particular, a single nucleotide polymorphism (SNP) can differentially affect different transcripts from the same gene. Moreover, SNPs potentially can have stronger effects than one might imagine by hitting a splice site or a site of post-translational modification in proteins; e.g. a splice SNP could result in removal of an entire exon. These features could be addressed by developing large datasets of protein isoforms and linking these against gene annotations [15,16].

c) *“Multi-dimensional Data Integration”.* All speakers and participants emphasized that proteomics data should be integrated with diverse biomedical information. Robert Gerszten, Massachusetts General Hospital, discussed the importance of connecting proteomics data with clinical measurements and metabolomics, and Gerstein emphasized the usefulness of connecting the protein networks with three-dimensional structures of proteins and protein complexes. This integration opportunity could be further pursued by solving co-crystal structures of proteins and using these to provide molecular details for interaction networks. Gil Omenn, University of Michigan, extended this comment by citing current work using I-TASSER algorithms to predict three-dimensional structures and conformations of pairs of splice variant proteins differentially expressed in Her2/neu breast cancer and infer the functional consequences of the sequence differences between the splice variants [17].

d) *“The Complexities and Subtleties of Detailed Integration”.* The challenges in achieving data integration in the framework of a working database system were underscored by Rolf Apweiler, European Bioinformatics Institute [18]. He pointed out that, in many instances, while one can get most of the integration done, there are some unresolved cases, including such major aims as connecting the genomics data from Ensembl to the proteomics information in Uniprot. Zhiping Weng, University of Massachusetts Medical School, highlighted how chromatin marks might be used to predict gene expression information in the framework of an integrative model. This approach represents moving beyond simply putting together the datasets to actually exploring how one dataset might be used to predict another.

### Quantitative proteomics by exploratory and targeted mass spectrometry methodologies (Matthias Mann, Max Planck Institute of Biochemistry, chair)

The four speakers (Matthias Mann; Joshua Coon, University of Wisconsin-Madison; Robert Moritz, Institute for Systems Biology; Forest White, Massachusetts Institute of Technology) addressed a mixture of technological, data-centric and functional biological proposals and issues. They emphasized the stunning technological progress in mass spectrometry-based proteomics [5-7,19-21]. Large-scale analyses corresponding to in-depth microarray and RNA-Seq methods for gene expression are now feasible. Since proteins are the workhorses of the cell, the capabilities of MS-based proteomics are crucially important to obtain a balanced view of the cell and to put genetic and genomic findings in biological context.

*“Expression Proteomics”.* Mann and Coon described recent and emerging innovations in shotgun proteomics. In contrast to studies limited to the most abundant dozens or hundreds of proteins in a specimen, the proteome can now readily be analyzed in great depth and with high quantitative accuracy. In yeast, nearly complete coverage of the expressed genes has already been achieved. Mann presented data obtained using the Orbitrap-Elite platform where more than 10,000 proteins were identified in a single lysate from a human HeLa cancer cell line [7] and from 10 other human cell lines, representing a broad range of organs of origin. Ruedi Aebersold’s laboratory has published similar deep quantitation of U20S osteosarcoma cells [6]. Comparison to deep sequencing (RNA-seq) data suggests that the majority of the functionally active proteome can already be quantified with such new technology [5-7]. However, to extend these capabilities to protein isoforms and to make them accessible to more laboratories, vigorous technology development should be pursued, using combinations of mass spectrometry methods and bioinformatics tools that detect post-translational modifications. Mann also presented a framework for intelligent data acquisition and real-time database searching using MaxQuant-Real Time, permitting searches according to specific GO terms (the example of the kinases activities was illustrated) and finding specific kinds of peptide modifications [22]. This capability is necessary for obtaining greater sequence coverage of the individual proteins, which would be helpful in distinguishing and mapping protein isoforms. Coon also described a smart data acquisition strategy in which the mass spectrometer is directed to identify specific peptides. Such strategies help to increase proteome coverage in shotgun experiments because they allow including important peptides for sequencing and excluding irrelevant ones. For proteins up to 50,000 molecular weight, top-down methods are becoming very useful [21]. Using this approach 3,000 different molecular species, representing about 1,000 main gene products, can be fully characterized by mass spectrometry in a single project. The application and further development of these approaches should be encouraged.

*“Targeted proteomics with selected reaction monitoring (SRM)”.* Robert Moritz described the SRM Atlas, a wholeproteome initiative jointly led by the Aebersold laboratory at ETH-Zurich and the Moritz laboratory at the Institute for Systems Biology in Seattle using the Triple-Quad mass spectrometers rapidly emerging from multiple manufacturers. In Selected Reaction Monitoring, several proteotypic peptides distinctive for each targeted protein are chosen for their expected transition properties in the mass spectrometer and then identified and quantified using corresponding heavy-labeled spiked-in peptides. Within just 2–3 years, multiple peptides for each of the expressed yeast genes and now 99% of the 20,300 human gene-coded proteins have been prepared; their mass spectra have been determined and shared publicly through the SRM Atlas. Proteins that are biomarker candidates from discovery phase research can be assayed with SRM peptides to facilitate experimental and clinical studies across a wide array of diseases. These peptide and spectra resources are valuable assets for the entire proteomics and life sciences research communities. This database of SRM peptide transitions can also be used as a reference to interpret experiments in which all peptides in a particular mass range are fragmented together. Measuring many of these proteins in complex specimens like tissue lysates or plasma will require further increases in sensitivity, using either anti-peptide antibodies [23] or enhanced mass spectrometry.

*“Post-translational modifications”. *Besides deep quantitative and targeted expression proteomics, an area of great promise is the large-scale identification, quantification, and mapping of post-translational modifications. Tens of thousands of phosphorylation, ubiquitinylation, acetylation, and glycosylation sites have been uncovered by mass spectrometry. Forrest White pointed out that we should now focus on the biological functions of these modifications, truly a grand challenge. Particular directions can include the mapping of kinase/substrate relationships using modified kinases, starting with specific pathways of particular interest in oncology. The same approaches will be applied to the entire array of protein classes in the long term. White emphasized the need for basic biochemical data, such as kD values, in order to better understand and model biological processes.

*“Protein Interactions”. *The area of protein interactions was discussed by many participants. Efficient approaches and protocols now exist for mapping interactions of full-length proteins. With quantitative proteomics, specific binders can be distinguished from background binders. Importantly, the specific interactions of modified peptides, DNA, RNA, and small molecules with their target proteins can now be addressed in a large-scale format. These represent important areas of biology and biotechnology where few alternative techniques exist.

The effect of individual genetic differences on the proteome (or lack thereof) has been the subject of a few pioneering studies but is still largely unexplored [24-26]. MS-based proteomics is uniquely positioned to measure the effects of these differences at the level where it counts, namely the level of protein expression or activity differences. This work will be essential to translate genetic differences to differences in pathways and differences in how those pathways should be modulated by drugs or other means.

### Study design and statistical challenges in clinical proteomics (Gilbert S. Omenn, University of Michigan, chair)

Omenn opened this session with comments about challenges in developing omics-based tests for cancers and other diseases. He emphasized that specification of the intended clinical use is the critical first step. Heterogeneity of etiology, pathogenesis, and responses to therapy among patients with identical diagnoses and heterogeneity within tumor masses provide major challenges for developing tests aimed at the clinical needs of diagnosis, prognosis, and guided therapy. Knowledge of mechanisms can enhance test development by providing a biological foundation for the test, rather than relying on statistical correlations. Integration of data from complementary gene expression, genomic, epigenomic, proteomic, and metabolomic platforms will enhance these complex studies. Finally, it should be acknowledged that it is a long path of discovery, confirmation, validation, clinical trials, and FDA approval to establish test validity and utility and gain reimbursement of the laboratory service.

Several major statistical challenges were identified: a) High-dimensional data with relatively few specimens tested in the discovery phase inevitably lead to high risks of over-fitting; an extreme case is two pooled specimens. b) Multi-site collection of specimens, with pre-analytical and analytical variation, generates prominent “lab effects” or “batch-effects”, which can overwhelm the disease associations; however, Nathan Price of the Institute for Systems Biology has emphasized the value of analyzing multiple laboratories results to estimate variance and find a common biomarker signature. c) There are always tradeoffs between sensitivity and specificity of test results, corresponding to type 1 and type 2 errors (false-positives and false-negatives); any claim of 100% sensitivity and 100% specificity should be viewed with maximal skepticism. A better parameter for a screening test is the positive predictive value (PPV), which takes account of the intended clinical use and the incidence of true positives in the population to be tested. d) Bias can be introduced in multiple ways (see below); for example, use of several equivalent methods, with selective reporting of the method that happens, perhaps randomly, to give the most favorable results. e) The variable ways of estimating false-positive rates in matching peptide sequences from mass spectrometry with protein databases; PeptideAtlas recommends a rigorous cutoff at 1% FDR (0.01) at the protein level, which generally corresponds to 0.16% (0.0016) at the peptide level [27].

Omenn concluded that new statistical methods and conventions are needed to enhance the integrated analysis of omics results from multiple platforms. Even if the data are collected from specimens on the same individual, compounding of errors and biases is likely. Biological knowledge of meaningful, testable pathways and networks should help in reducing biases.

Steven Skates, Massachusetts General Hospital, presented “Study Design in Omics Biomarker Research”. He discussed clinically-derived quantitative goals and sources of bias that are threats to the validity of omics-based biomarkers. It is common to characterize tests by sensitivity (proportion of true positives detected) and specificity (proportion of false-positives). It is necessary to optimize the combination in light of prevalence of the condition to be detected and clinical and ethical importance of missing the diagnosis (false-negatives) or making a false diagnosis (false-positives). A judgment about benefits of true positive and true negative and harms of false positive and false negative results is needed [28]. Perhaps this can be done by stating a minimum benefits/harms ratio, with input from clinicians. Skates’ example of early detection of ovarian cancers (prevalence 1 in 2500 postmenopausal women) showed the impact of introducing a confirmatory test, like ultrasound, after the molecular screening test. How many patients would have to undergo testing and then, of those testing positive, surgery to find one case of ovarian cancer? What would be an acceptable ratio? For example, to achieve a ratio of five surgeries to one patient with ovarian cancer would require test specificity of 98% (2% false positives = 50/2500) plus the 10-fold benefit of ultrasound. He then outlined the stories of OvaCheck and OvaSure as tests that failed due to bias. The intended use for the FDA cleared OVA1 was much narrower, “to assess the likelihood of malignancy in patients with ovarian adnexal mass when surgery is planned and not yet referred to an oncologist”. He concluded that investigators should avoid biased early studies, which set us off in wrong directions, as it would be more efficient and more scientifically sound to seek high-quality, clearly-unbiased specimens for early stage studies.

Lisa McShane, National Cancer Institute, presented "Statistical Issues in the Development of Reliable and Clinically Relevant Prognostic and Predictive Proteomic Signatures”. She discussed practical methods to operationalize classifiers, risk scores, or decision trees as mathematical models for molecular markers, whether RNA, DNA, or proteins [28,29]. It is critical to define the intended use across the categories of early detection or risk estimation before there is a clinical diagnosis. These categories include confirmation, staging, and subtyping upon diagnosis; prognosis or prediction before the start of therapy; desired responses and potential toxicity from therapy; and post-treatment outcomes, including survival and absence or recurrence of disease. Predictive signatures refer to treatment effect modifiers. Prognostic effects are typically quantified by hazard ratio, while predictive effects are typically quantified by ratio of subgroup-specific treatment hazard ratios. The goal is to create and validate a clinical test from molecular data, as has been done with the 21-gene recurrence risk score (OncotypeDX) and the Mammaprint 70-gene signature, which are used clinically to identify women with such low risk of metastasis that adjunct chemotherapy can be considered unnecessary. She demonstrated how to answer such questions as: Is the prognostic information sufficiently strong to influence clinical decisions? Does the predictor provide information beyond standard prognostic factors (i.e., “added value”)? Proper control groups are critical for interpreting results with and without use of the marker. Data from randomized clinical trials can distinguish benefit of therapy only for marker-positive participants from benefit for all participants (in which case the marker test may not add value). The process requires multiple steps, including lock-down of the assay and classifier, then internal validation on suitable specimens, and then external validation on independent set(s) of specimens/data. Expert statistical steps involve feature selection and supervised dimension/data reduction. Over-fitting is a particularly devastating problem, which is predictable; when there are too many parameters relative to the number of specimens or patients, the model will describe random error or noise instead of an underlying relationship. Leave-one-out and other cross-validation methods also must be done expertly. Statistical maneuvers cannot overcome built-in biases from variable specimen handling and other lab or batch effects.

The speakers reinforced the importance of identifying pathways and networks that make biomarker candidates biologically meaningful and credible. There is a special opportunity for building on NIH Common Fund programs such as the Technology Centers for Networks and Pathways and help frame the Biology- and Disease-driven components of the global Human Proteome Project (B/D-HPP) being launched by the Human Proteome Organization [3]. This would effectively leverage the tremendous investments around the world that have already been made in the mass spectrometry, protein –capture reagents, knowledgebase pillars for the HPP, and in the HPP chromosome-centric program.

### Proteomic technologies in a clinical setting (Daniel W. Chan, Johns Hopkins University School of Medicine, chair)

Chan discussed the translation of proteomic technologies into a clinical setting [30-32]. He presented multiple reasons for the significant gap between biomarker discovery, validation and translation. The development of OVA1, the first proteomic IVDMIA (*in vitro* diagnostic multivariate index assay) cleared by the FDA, was described to illustrate the concept of “the four bridges for biomarker translation”: 1) clearly define a specific clinical “intended use” (unmet clinical needs); 2) generate sufficient evidence in preliminary studies to justify the investment for a large-scale validation trial; 3) select/develop assays with performances suitable for clinical use; and 4) conduct a pivotal clinical trial to demonstrate clinical utility to obtain regulatory approval and to gain acceptance by the clinical community [31]. Chan proposed a roadmap for the translation of proteomics technologies into clinical settings. The roadmap requires close collaboration between researchers, industries, regulators, clinical chemists and clinicians, including private-public partnerships, to leverage existing NIH programs such as the NCI Clinical Proteomics Tumor Analysis Consortia (CPTAC) and Early Detection Research Network (EDRN) and the NHLBI, NIDDK, and NIAD clinical proteomic programs. Dr. Chan suggested that such a joint effort would accelerate the development of clinically useful technologies and biomarkers and make a significant impact to fulfill the unmet clinical needs for patients’ personalized health care.

Barry Dowell, Abbott Laboratories, pointed out key challenges in biomarker commercialization: the selection of biomarkers to address unmet needs, pre-analytical and analytical issues, clinical performance assessment, regulatory approval, physician education, launch of new products, post-launch studies, and marketing issues. These factors are all important to bring a new product (biomarker) to market. Key considerations for a successful product include clinical utility, reagent availability, performance characteristics, patent status with freedom to operate, and licensing terms. He emphasized pre-analytical considerations of specimen collection, biomarker stability and its specific forms in blood, as well as biological variability. Analytical performance depends on establishing assay design requirements, identifying key reagent components, and optimizing test procedures and manufacturing processes. Clinical performance requires establishing specimen collection Standard Operating Procedures and study designs with sufficient statistical power. FDA submissions should use multi-site studies with appropriate patient specimens for the specific “intended use”. Finally, companies marketing new biomarkers face different regulatory and re-imbursement processes from country to country, competition from multiple biomarkers, and the need for clinical studies to convince the medical community of the value of the new biomarker.

Darryl Palmer-Toy, Southern California Kaiser Permanente, presented a clinical laboratory practitioner perspective on proteomic biomarker discovery. Taking a biomarker from the research laboratory into the routine clinical laboratory requires proactive three-way collaboration involving the research lab, the diagnostics industry and the clinical laboratory [29]. He stated that it’s “a jungle out there” in clinical labs, and “shiny new biomarkers quickly lose their luster”. He pointed out the importance of properly collected patient specimens to obtain correct analytical results and correct clinical decisions. An ideal assay should be tough enough to stand up to abuse. Sometimes, consistent results are more important than “true” results (e.g. the hemoglobin A1C test for monitoring diabetes). Diagnostic tests should provide clinically useful information not available by other means and at a reasonable cost, including quality control.

Maria M. Chan, Food and Drug Administration, gave the FDA perspective on proteomic biomarker/technology translation. FDA regulates *In Vitro* Diagnostics (IVDs) including reagents, instruments and systems using human specimens. FDA uses a risk-based classification system based on the risk to the patient due to false results: class I with low risk is exempted; class II with medium risk requires 510(k) pre-market notification; class III with high risk requires Pre-Marketing Approval (PMA). “Intended Use” determines the FDA classification, the review path and the type of study required. The basis of device review by FDA is the balance between safety and effectiveness. All IVDs must have adequate analytical and clinical performances and meet labeling requirements on, intended use, warnings, limitations, interpretation of results and performance summary. For proteomics IVDs, one should use patient specimens with results spanning entire concentration ranges; the performance at the cut-off value is critical. Precision, limit of detection, specificity, matrix effect, accuracy, stability and pre-analytic variables are performance criteria. Clinical validation should include a study design with target populations from a minimum of 3 sites, sample size justification, patient selection criteria and a pre-specified hypothesis. Other pre-study considerations include appropriate statistical plans, with the training set different from the validation set; consideration of possible confounding co-variables; and completion of analytical validation preceding clinical validation. She recommended a Pre-IDE as a very useful tool for a company to obtain free protocol review by FDA and to gain advice on regulatory process and feedback on proposed studies. This will prevent unnecessary waste of time and resources. Finally, she mentioned new FDA draft guidance for companion diagnostics (issued 12 July 2011) and for research-only or investigation-only (RUO and IUO) products (issued 1 June 2011), as well as several IVDs cleared by the FDA for leukemia, breast, ovarian, prostate and lung cancers.

### Conclusions from general discussions

A session within the workshop was fully dedicated to discuss potential scientific opportunities and identify compelling ideas for future developments in the field of proteomics. Several potential concepts were discussed and considered in the context of the presentations by the speakers. After evaluation of several important and valuable opportunities, the idea of generating a human proteome network emerged as the most compelling opportunity. However, it was also emphasized that, it would be important to further support technology development aimed at a more comprehensive characterization of the proteome, further develop and support proteomic data resources, and work toward an inter-agency roadmap that would facilitate the translation of proteomic discoveries.

### Human proteome networks in health and disease - A major scientific opportunity

The Human Genome Project was initiated almost 25 years ago. Its findings and its approaches have transformed much of biomedical research and clinical genetics practice. Connecting genomics knowledge to phenotypes is critical for common diseases; research on functional genomics and gene/environment interactions requires understanding and assays of proteins and protein networks. The genome parts list has inspired a corresponding approach to the identification and characterization of protein products; rapid progress recently has brought us to antibody evidence and immunohistochemical tissue localization of ~12,000 of the ~20,000 predicted protein gene products in the Human Protein Atlas and mass spectrometry-based evidence in SwissProt/UniProt for about 13,000 of the ~20,000 protein products. Completion of the protein parts list is the primary goal of The Human Proteome Project, using both chromosome-centric and biology/disease-driven approaches [3]. That leaves enormous work to be done on protein isoforms, dynamic regulation of protein expression, and interactions of proteins with macromolecules and small molecules critical to cellular and organismal function.

Projecting ahead 25 years, the participants of this Workshop envision that one of the most promising outcomes of this modern biomedical research, and especially a focus on protein networks, could be the transformation of health care into a predictive, preventive, personalized and participatory system of care (“P4 medicine”) [33]. Key pillars of P4 medicine are emerging from omics-based research and the field of systems biology. Interpretation of complete personal genome sequence data will require a much better knowledge of how gene products encoded by the human genome interact with each other to contribute to complex molecular interactome networks and cellular systems that underlie the biology of our tissues and organs. Extensive understanding of the functional, dynamic and logical relationships taking place in the context of complex interactome networks will eventually drive two major aspects of P4 medicine: integrated biomarker discovery and systems pharmacology. Biomarker discovery will improve the predictability of specific diseases by integrating personal genomics information, knowledge of environmental components and understanding of the properties of cellular systems. Similarly, safer and more predictably effective personalized therapies will emerge from understanding of complex relationships between proteins and cellular networks.

What became increasingly clear throughout this Workshop is that one critical component missing for this vision to eventually become reality is a freely-available global map of the human proteome in terms of macromolecular interactions between its components, i.e. a wiring diagram of functional relationships between genes and gene products. We see the development of a Human Protein Network as a major scientific opportunity. A nearly complete map of human protein-protein, protein-nucleic acid, and protein-small molecule interactions could be generated and this information could be combined with biologically-driven findings to complete the human proteome parts list and functional networks for the ~20,000 protein-coding genes and their products. Comparative analyses in model organisms would enhance the human studies. One of the major outcomes of such interactome maps would be a wiring diagram that could be used to make sense of complex traits starting from currently available Genome-Wide Association studies (GWAS), at least those variants producing non-synonymous mutations in protein-coding genes, cancer genome sequencing efforts such as The Cancer Genome Atlas (TCGA), and key consortia such as ENCODE. Interactome network maps would serve as foundational information for systems biology, enhance understanding of the pathways between genotypes and phenotypes, and improve the predictive power of integrated and personalized biomarkers, therapies, and combinations of therapies.

Models exist as to how one could organize a global large-scale study, inspired by the Human Genome Project and its many follow-on initiatives. In terms of the specific needs to address the dynamic aspects of proteome function, rather than the linear and binary information in DNA sequences, we find inspiration in the example of the ENCODE consortium. ENCODE uses defined cell lines to generate high-throughput unbiased systematic datasets and maps of protein-DNA interactions, but leaves biological studies and functional follow-ups to other granting mechanisms.

A Network Biology/Interactome Mapping Project can be defined in terms of end-point goals and intermediate milestones using empirical frameworks. For a comprehensive human binary protein-protein interactome network, it has been established that on the order of ~150,000 interactions are to be found in what will constitute the “Reference” interactome network [34]. At this stage, the combination of low-throughput and high-throughput datasets curated by databases such as BioGrid (see above) indicate that the community has assembled about 20% of that number of high quality interactions of the Reference interactome network. In other words approximately 80% of the interactome remains to be mapped.

Static maps of macromolecular interactions need to be combined with network-based datasets consisting of other types of functional and dynamic relationships between genes and gene products, such as: i) protein expression data by measuring precisely the proteome content of particular cell lines and tissues, as demonstrated elegantly by Mann during the Workshop, ii) tissue and subcellular localization data obtained using both mass spectrometry and immunohistochemistry, iii) kinase-substrate or other post-translational enzyme-target relationships as measured by protein arrays, and then iv) translation of the corresponding gene-gene network information into a better description of tissue lysates and body fluids for integrated biomarker discovery efforts.

In terms of future directions, it will be crucial to organize this map such that it can be used to generate dynamic models that can be integrated with both Mendelian and complex multifactorial diseases. It will be extremely important to democratize access to peptide and protein data, interactome networks data, and powerful analytical platforms. The Workshop participants agreed that it is feasible to generate a human macromolecular interaction map with the technology that is available today. However, developing new technology platforms for proteomic analysis is needed to better characterize the heterogeneity of tissues and tumors.

In summary, a major obstacle to being able to move forward with integrated biomarker discovery and systems pharmacology is the generation of a high quality, freely available and nearly complete map of the human interactome network. This rapidly emerging scientific challenge is also a great scientific opportunity. Such a project would benefit from and go beyond the example of the ENCODE consortium, particularly in terms of shared goals by the participants and shared quality standards to map the interactome network. A protein interactome networks effort would build on previous and current trans-NIH Common Fund investments, including the National Centers for Biocomputing, the Technology Centers for Networks and Pathways, the Library of Integrated Network-based Cellular Signatures, the Protein Capture Reagents, and the Interdisciplinary Research Consortia. If implemented to a sufficiently large extent this interactome mapping project could have a high impact by producing a systematic, unbiased, freely available wiring diagram for a systems biology-based implementation of P4 medicine.

## Competing interests

The authors declare that they have no competing interests.

## Authors' contributions

MV, DWC, MG, MM, GSO, DT, and SS participated in writing the manuscript and in discussing its content. All authors listed under WP participated in the discussions that led to the writing of this report. All authors read and approved the final manuscript.

## Workshop participants

Rolf Apweiler, European Molecular Biology Laboratory, European Bioinformatics Institute; Joel S. Bader, The Johns Hopkins University; Stephen Barnes,, University of Alabama at Birmingham; Raija Bettauer, Bettauer BioMed; Maria M. Chan, U.S. Food and Drug Administration; Joshua Coon, University of Wisconsin, Madison; Kara Dolinski, Princeton University; Barry L. Dowell, Abbott Laboratories; Charles G. Edmonds, National Institute of General Medical Sciences; Suzanne Gaudet, Dana-Farber Cancer Institute, Harvard Medical School; Robert Gerszten, Massachusetts General Hospital; Jacob Kagan, National Cancer Institute; Sanford P. Markey, National Institute of Mental Health; Richard Mazurchuk, National Cancer Institute; Lisa McShane, National Cancer Institute; Robert L. Moritz, Institute for Systems Biology; Darryl Palmer-Toy, Kaiser Permanente; Akhilesh Pandey, The Johns Hopkins School of Medicine; Peipei Ping, University of California Los Angeles; Malu Polanski, Kelly Services; John Quackenbush, Dana-Farber Cancer Institute, Harvard School of Public Health; Douglas Sheeley, National Institute of General Medical Sciences; Steven Skates, Dana-Farber Cancer Institute, Harvard University, Massachusetts General Hospital; Sudhir Srivastava, National Cancer Institute; Wendy Wang, National Cancer Institute; Zhiping Weng, University of Massachusetts Medical School; Forest White, Massachusetts Institute of Technology.
